# Adherence to the Mediterranean Diet in Children and Adolescents and Association with Multiple Outcomes: An Umbrella Review

**DOI:** 10.3390/healthcare12040449

**Published:** 2024-02-09

**Authors:** Alice Masini, Laura Dallolio, Francesco Sanmarchi, Francesco Lovecchio, Mario Falato, Yari Longobucco, Marcello Lanari, Rossella Sacchetti

**Affiliations:** 1 Department of Translational Medicine, University of Eastern Piedmont, 28100 Novara, Italy; alice.masini@uniupo.it; 2 Department of Biomedical and Neuromotor Sciences, University of Bologna, 40126 Bologna, Italy; laura.dallolio@unibo.it (L.D.); francesco.lovecchio3@studio.unibo.it (F.L.); mario.falato@studio.unibo.it (M.F.); 3 Department of Health Sciences, University of Florence, 50134 Firenze, Italy; yari.longobucco@unifi.it; 4 Pediatric Emergency Unit, IRCCS Azienda Ospedaliera Universitaria di Bologna, 40126 Bologna, Italy; marcello.lanari@unibo.it; 5 Department of Education Studies “Giovanni Maria Bertin”, University of Bologna, 40126 Bologna, Italy; rossella.sacchetti@unibo.it

**Keywords:** Mediterranean Diet, children, adolescents, health effects

## Abstract

The Mediterranean Diet (MD) has been linked to numerous health benefits. This umbrella review aims to synthesize evidence from systematic reviews on the MD’s impact on children and adolescents aged 6 to 19 years. Following Joanna Briggs Institute guidelines, we included 11 systematic reviews focusing on the MD’s effects on health outcomes, such as asthma, anthropometric measures, physical fitness, and inflammatory markers. The results indicate a protective role of the MD against childhood asthma due to its antioxidant-rich components. However, evidence for its impact on allergic conditions like rhinitis and eczema is inconclusive. Findings regarding anthropometric outcomes, particularly BMI, are limited and inconsistent. A positive correlation was observed between MD adherence and increased physical activity, improved physical fitness, quality of life, and reduced sedentary behavior. Additionally, the MD showed an inverse relationship with pro-inflammatory biomarkers, suggesting anti-inflammatory benefits. The inconsistency in BMI findings and the potential anti-inflammatory properties of the MD warrant further longitudinal research. Future studies should explore the MD’s impact on cognitive functions, academic performance, and mental health in this age group, highlighting the significance of establishing healthy eating habits early in life.

## 1. Introduction

The Mediterranean Diet (MD) has traditionally been consumed in countries that surround the Mediterranean Sea (e.g., Greece, Spain, and Italy). The United Nations Educational, Scientific, and Cultural Organization (UNESCO) has recognized the long history of this diet and in 2010 classed the MD among the list of intangible cultural heritages of humanity [1]. The objective of this initiative was to safeguard the immense legacy representing the cultural value of the Mediterranean Diet and to share its values and benefits internationally. The concept of the MD is better described as a lifestyle model characterized by a healthy dietary pattern that includes a high intake of plant foods comprising mainly fruits and vegetables, whole grains, legumes, nuts, and seeds; locally grown, fresh, seasonal, unprocessed foods; olive oil as a main cooking ingredient and source of fat; low to moderate amounts of cheese and yogurt; low quantities of meat and meat products and higher quantities of fish; and low to moderate amounts of red wine with meals for adults [2].

This diet has been widely recognized for its numerous health benefits, including a significant reduction in overall mortality and in morbidity and mortality from cardiovascular diseases and other major chronic diseases [3,4,5,6,7,8]. Many components and individual foods of the Mediterranean Diet are beneficial to health (for example, extra virgin olive oil), but overall, it is the combination of foods that is linked to improved health [9]. At the same time, the MD represents the best model of a sustainable diet because it has positive effects on the socio-cultural, economic, and environmental spheres [10]. 

The MD was recognized in 2019 as a possibly universal model of a healthy diet from the EAT-Lancet Commission [11,12]. Precisely for this reason, it is promoted worldwide as one of the healthiest dietary patterns [13].

Building on this foundation, it is imperative to consider specific nutritional needs and the potential impact of dietary choices during the early stages of life. Childhood and adolescence are not merely transitional phases but rather pivotal periods that shape future health outcomes [14]. The dietary patterns adopted during these formative years lay the groundwork for lifelong eating habits, and they influence the risk profile for various non-communicable diseases. Hence, the exploration of the MD within the context of early life not only enriches our understanding of its comprehensive benefits but also aligns with global efforts to instill sustainable, health-promoting dietary practices from a young age. This consideration seamlessly bridges the established virtues of the MD as a model for adults to its consequential role in nurturing the younger generation, setting the stage for a detailed examination of its implications for children and adolescents. Eating healthy during childhood and adolescence is fundamental to growth, cognitive development, overall well-being, and school performance [15]. Adherence to the MD is defined through scores that estimate conformity to the dietary pattern of the studied population.

Globally, there has been a reduction in the adherence to the MD from the 1960s [16]. In regards to children and adolescents from Mediterranean countries, the overall rate revealed that 21% of the surveyed population had low adherence to the MD versus 10% with high adherence in the last years [17].

At the same time, the prevalence of obesity, type 2 diabetes, and other diet-related health issues among children and adolescents has been rising globally, emphasizing the need for effective dietary interventions [18]. Knowledge of the benefits of the MD for children and adolescents could be valuable for promoting adherence among these populations.

Despite the growing body of literature on the benefits of the MD, there is a lack of comprehensive evidence synthesis focusing on children and adolescents. Meanwhile, regarding adults, there is a recent umbrella review conducted by Sofi et al. that finds robust evidence for reductions in overall mortality, cardiovascular diseases, overall cancer incidence, neurodegenerative diseases, and diabetes [8]. As regards children and adolescents, some research has investigated the impact of the Mediterranean Diet on various health outcomes, including physical and mental health, cognitive functioning, academic achievement, and quality of life [19,20]. However, the findings are often scattered across individual studies and systematic reviews, leading to potential inconsistencies and knowledge gaps.

Numerous indices have been developed to estimate adherence to the MD, reflecting its recognized importance in promoting health. However, the literature reveals a methodological challenge: the heterogeneity of these indices. The variation in dietary indices, encompassing diverse food components and scoring systems, complicates the comparability of studies and the consolidation of findings. This methodological diversity can potentially cloud our understanding of the true relationship between MD adherence and health outcomes. It is crucial to note that the inconsistency in measurement tools does not negate the substantial evidence supporting the positive influence of the MD on health. Rather, it highlights the need for methodological refinement and standardization in assessing MD adherence to ensure the reliability and clarity of future research in this domain.

Given the need for a more comprehensive understanding of the evidence, an umbrella review presents an ideal methodological approach. Umbrella reviews synthesize the evidence from existing systematic reviews and meta-analyses, providing a more reliable and exhaustive overview of the literature [21]. To date, no umbrella review has been conducted to specifically assess the benefits of adhering to the Mediterranean Diet among children and adolescents. By conducting an umbrella review specifically targeting children and adolescents, we aim to address these gaps and provide a clearer understanding of the available evidence.

The primary objective of this umbrella review is to investigate the evidence regarding the benefits of adhering to the Mediterranean Diet in terms of physical and mental health outcomes, cognitive functioning, academic achievement, and quality of life among school-aged children and adolescents from 6 to 19 years old.

Our umbrella review will uniquely contribute to the field by synthesizing the current state of evidence and providing a comprehensive and reliable overview of the benefits associated with the Mediterranean Diet in this age group.

This knowledge could guide the development of future interventions, inform clinical practice, and shape public health policies aimed at promoting healthy eating habits among young people.

## 2. Materials and Methods

The present umbrella review was conducted in accordance with the Joanna Briggs Institute Umbrella Review Methodology [22]. The review protocol was registered in the PROSPERO Database for Review (Prot. CRD42023408806).

### 2.1. Inclusion and Exclusion Criteria

Inclusion and exclusion criteria were established based on the PICO strategy (Table 1).

For inclusion, we considered systematic reviews and meta-analyses that examined populations of children and adolescents aged 6 to 19 years. These studies needed to specifically explore the relationship between adherence to the Mediterranean Diet and various outcomes, encompassing aspects including physical and mental health, cognitive functioning, academic performance, and overall quality of life.

On the contrary, certain studies were systematically excluded from our analysis to maintain the specificity and quality of our review. These excluded studies encompassed those not authored in English, as well as scoping or narrative reviews, which did not align with our methodological focus. Additionally, we omitted studies that did not specifically assess adherence to the Mediterranean Diet. Lastly, to maintain a targeted age demographic, studies focusing on populations outside our age range of interest, specifically those including kindergarten children or adults over the age of 19 years, were also excluded.

### 2.2. Search Strategy

Two reviewers (AM and YL) independently performed a comprehensive literature search in PubMed (Medline), the Cochrane Library, Embase, PsychINFO, CINAHL (EBSCO), and Scopus until December 2022 while intentionally not specifying a start date. Additional studies were identified by examining the reference lists of the located articles. No time limitation was applied.

The following search terms were employed across the different databases, with adaptations made based on the specific language requirements of each database. (Mediterranean Diet OR Diets, Mediterranean OR Diet, Mediterranean OR Mediterranean Diets OR (“Diet, Mediterranean”[Mesh])) AND ((“Child”[Mesh]) OR (“Adolescent”[Mesh])) AND (systematic review OR meta-analysis)). Furthermore, the gray literature was explored through manual searches of key conference proceedings, journals, and professional organizations’ websites. Lastly, the references list of included primary papers were screened, following a snowball technique to identify additional eligible papers.

### 2.3. Assessment of Methodological Quality

Five reviewers independently and blindly assessed the risk of bias for the potentially included studies following the JBI critical appraisal instrument for Systematic Review [22]. Disagreements between reviewers were resolved through discussion and, if necessary, the involvement of a tiebreaker.

The JBI checklist [22] comprises eleven items designed to evaluate different methodological aspects of a systematic review and/or meta-analysis, including the appropriateness of the search strategies (i.e., clear PICO question, adequate number of databases searched, and clear search terms), the approach to evidence synthesis (i.e., data extraction methodology), potential sources of biases, and implications for future studies and policy making (i.e., strength of the evidence, limitations, future directions). Decisions about a scoring system were made in advance and agreed upon by all reviewers before starting critical appraisal.

Reviews were awarded one point for each criterion met. The overall score of a study could range from zero to eleven. In accordance with previous studies [23], three cutoff points were established: studies scoring zero to four points were classified as having a high risk of bias, five to seven points indicated a medium risk, and eight to eleven points signified a low risk of bias, respectively.

### 2.4. Data Collection

The review team independently and blindly screened all eligible articles’ titles and abstracts using the PICO criteria, with each study being screened twice by different reviewers. After duplicate removal, full texts of all potentially eligible studies were obtained, extracted, and independently reviewed by the reviewers (AM, FS, FL, MF, LD, and RS) utilizing a pretested data extraction form. To ensure rigor and consistency, each article was independently assessed by two reviewers. Inclusion disagreements were resolved through discussion among the research team.

Data from the included studies were extracted by the researchers using JBI data extraction tools [22]. Retrieved details encompassed the first author’s name and country, the publication year, characteristics of the studied populations, the review’s type, outcomes measures (i.e., asthma and allergies, anthropometric variables, healthy habits and physical fitness, clinical markers, quality of life, and other outcomes), methods of Mediterranean Diet adherence assessment, overall results, and strengths and limitations of the review.

If a systematic review contained studies outside of the present umbrella review’s PICO scope, only the results aligning with our inclusion/exclusion criteria were extracted when possible.

To ensure a thorough and accurate data extraction process, several quality assurance measures were implemented. Prior to extraction, a data extraction form was developed and piloted on a subset of included studies. This helped the reviewers to familiarize themselves with the extraction process, identify any potential issues, and refine the form as needed. Once the form was finalized, the reviewers (AM, FS, FL, MF, LD, and RS) independently extracted data from the included studies, with each study being reviewed by at least two reviewers to ensure accuracy and consistency.

To further enhance the reliability and consistency of data extraction, a calibration exercise was conducted among the reviewers. During this exercise, each reviewer independently extracted data from a common set of studies using the finalized data extraction form. The results were then compared, and discrepancies were discussed to clarify any misunderstandings or ambiguities. This process helped to establish a common understanding of the extraction process and ensure consistency among reviewers.

Throughout the data extraction process, regular meetings were held among the research team to address any questions or concerns that arose. This allowed for continuous quality control and facilitated the resolution of any uncertainties or disagreements.

## 3. Results

**Figure 1:**
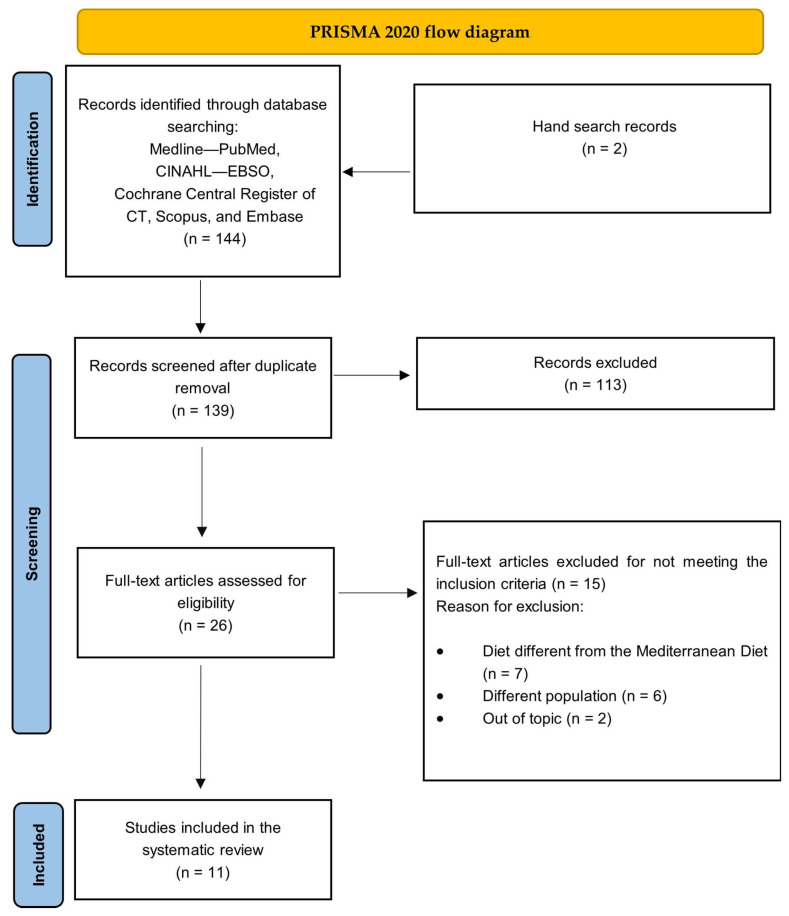
PRISMA flowchart.

As shown in Figure 1, a total of *n* = 144 systematic reviews were retrieved from the searched databases and through the hand search. After duplicates were removed, we screened *n* = 139 abstracts. The research team screened all of the potential full text papers (*n* = 26), and, finally, a total of *n* = 11 papers [24,25,26,27,28,29,30,31,32,33,34] met the inclusion criteria, as described in Figure 1. Among a total number of 11 systematic reviews included in the present umbrella review, only 2 papers included a meta-analysis [25,28]. The years of publication ranged from 2013 to 2022, with most of the papers published from 2020 to 2022. The geographic origin of the studies was Spain (*n* = 3 [25,28,30]), Portugal (*n* = 1) [26], Australia (*n* = 2 [27,33]), Greece (*n* = 1 [29], Peru (*n* = 1 [24]), Italy (*n* = 1 [34], the USA (*n* = 1 [31]), and Iran (*n* = 1 [32]).

The characteristics of the included systematic reviews were heterogeneous. The reviews included a total sample size ranging from 5661 to 234,236. The target groups of the eligible studies ranged in ages between the different reviews, although our work was focused only on children and adolescents from 6 to 19 years old. Most of the studies included in the reviews used a cross-sectional study design. All of the included reviews’ investigated populations lived in both Mediterranean and non-Mediterranean countries.

The studies reported different tools to evaluate adherence to the Mediterranean Diet. Among these instruments, the most commonly used was the MD Quality Index for children and adolescents (KIDMED) [35].

However, many variants of KIDMED were used (i.e., exclusion of items relating to fast food consumption or breakfast). Additionally, the Mediterranean Diet Score (MDS), developed by Trichopulou [36], was used in many studies, including some variations of the original version. Finally, the Krece Plus test [37], the Mediterranean lifestyle index (MediLIFE Index) [38], or an ad hoc score were rarely used.

**Figure 2:**
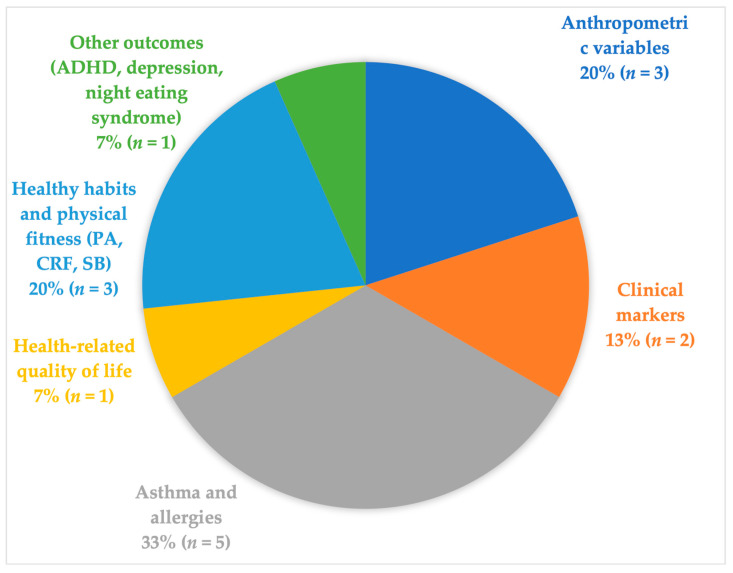
Map of the main categories of outcomes associated with MD adherence.

The 11 included systematic review investigated the association between MD adherence and 65 parameters, which we classified into six main categories (Figure 2 and Table 2). Some reviews analyzed multiple outcomes and were thus included in several of the following six primary categories.

### 3.1. MD, Asthma, and Allergies

Four systematic reviews [26,29,31,33] and one meta-analysis [25] specifically investigated the association between the MD and asthma. Overall, all of the studies indicated a protective role of MD adherence against childhood asthma but not for allergic rhinitis or eczema. Moreover, Garcia Marcos et al. [25] confirmed this inverse significant association with a meta-analysis between MD adherence and SCW, severe current wheeze (OR = 0.66, IC 0.48, 0.90) and between the MD and current wheeze (OR = 0.85, IC 0.75, 0.98).

### 3.2. MD and Anthropometric Variables

The anthropometric variables included body mass index (BMI), waist circumference (WC), adiposity, obesity, body fat mass, skinfold, and neck circumference. Three systematic reviews [26,30,34] evaluated the impact of MD adherence on anthropometric variables. In general, there is only limited evidence of the value of the MD for maintaining a healthy body weight, and no consensus on BMI. The other outcomes included in the anthropometric categories presented an inconsistent association with MD.

### 3.3. MD, Healthy Habits and Physical Fitness

Healthy habits were monitored by assessing physical activity (PA) levels and sedentary behavior (SB). Physical fitness was monitored through cardiorespiratory fitness (CRF), speed and agility, and muscular fitness. Two studies investigated the association between the MD and physical activity (PA) levels. García-Hermoso et al. 2022 [28] and Iaccarino et al., 2022 [34] found a direct relation between the MD and PA monitored through the use of actigraph accelerometers and/or questionnaires.

In particular, the meta-analysis conducted by Garcia-Hermoso statistically confirmed the association between the increase in PA levels and adherence to the MD (r = 0.14, IC 0.11, 0.17) [28].

Sedentary behavior was investigated in two studies [28,34]. Iaccarino et al. [34] underlined a negative association between SB and the MD, like Garcia Hermoso et al. [28], who reported the same inverse association confirmed by the meta-analysis (r = −0.15, IC –0.20, −0.10).

Three studies [28,32,34] investigated the association between the MD and cardiorespiratory fitness (CRF). The review conducted by Omid-Eslami et al. [32] reported that eligible studies confirmed that MD adherence was directly associated with CRF improvement. Iaccarino et al. [34] found a positive association between the MD and CRF only in one eligible study. Finally, Garcia-Hermoso et al. [28] in the meta-analysis confirmed a direct relationship between the MD and CRF (r = 0.22, IC 0.13, 0.31).

### 3.4. MD and Clinical Markers

The systematic review conducted by Bujtor et al. [27] showed an inverse association between MD adherence and pro-inflammatory biomarkers. Most studies have examined the effect of the MD on CRP, IL-6, and TNF. For instance, Teixeira et al. [26] found a positive association between greater adherence to the MD and lower blood CRP concentrations. This result was based on only one cross-sectional study included in their review.

### 3.5. MD and Quality of Life

Only a systematic review [24] based on nine studies investigated the association between the MD and health-related quality of life, finding overall a positive association with the general score and species subdomain, including physical wellbeing, peers, and school environment.

### 3.6. Other Outcomes

One systematic review included studies focused on the association between the MD and different outcomes that could not be included in the aforementioned main categories. Teixeira et al. [26] included one article focused on MD adherence and bone mineral density, three articles on blood pressure, one article on albuminuria, and one article on depression and night eating syndrome. However, one article included in Teixeira et al.’s [26] work investigated the association between MD adherence and ADHD syndrome, finding an inverse significant association.

### 3.7. Quality Assessment Results

Table 3 summarized the risk of bias score of the included reviews. Among 11 reviews, 7 were scored as having a low risk of bias due to the sound methodological structure. Only four reviews were scored as having a medium risk of bias, mainly due to the lack of publication bias analysis, clear inclusion/exclusion criteria, and critical appraisal conducted by two or more independent reviewers. Table S1 explains in detail the assessment of each domain using the JBI tool [19].

## 4. Discussion

### 4.1. Main Results: Overview

The present review is the first umbrella review summarizing the evidence of 11 systematic reviews investigating the association between the benefits of adhering to the MD and different outcomes among children and adolescents. Overall, the present umbrella review provided evidence regarding the effects of adherence to the MD and asthma, physical fitness, inflammation markers, and quality of life.

### 4.2. Asthma and Allergies

Starting from asthma and allergies, the findings suggested an inverse association. As confirmed by a recently published systematic review [39] that aimed to underline the effects of a healthy diet on asthma and wheezing in children and adolescents, the effect of the MD on asthma was more prominent. The Mediterranean Diet has been linked to a lower risk of asthma in numerous epidemiological studies [40]. Fruits and vegetables contain a wealth of antioxidants and various bioactive elements that contribute to the maintenance of lung function. These antioxidants serve as dietary agents that stimulate lung tissue as a reaction to oxidative stress, thereby minimizing the harm to respiratory systems from reactive oxygen radicals [41]. Due to the fact that fruit and vegetables are rich in antioxidants, they can reduce inflammatory reactions and asthma symptoms and improve lung function. Consuming fish, most of which are high in long-chain n-3 polyunsaturated fatty acids, has been demonstrated to effectively decrease inflammatory responses and alleviate asthma symptoms [42]. At the same time, the effectiveness of cereals in combating asthma remains somewhat unclear; however, it is believed that whole grains could offer protection against asthma by leveraging the antioxidant and anti-inflammatory properties of their components (e.g., vitamins, minerals, and phytonutrients), but more studies should be performed in order to understand the mechanism. However, to date, no evidence can be identified for allergic rhinitis, eczema, or atopy. It is important for clinicians to note this distinction. While the MD has shown potential benefits in various health outcomes [43], its impact on allergic conditions remains inconclusive. Allergic rhinitis, eczema, and atopy are multifactorial conditions with a combination of genetic and environmental triggers [44]. Dietary patterns, including the Mediterranean Diet, may influence some aspects of these conditions, but it is evident that they are not the sole determinants. These findings emphasize the necessity for comprehensive management strategies for patients with these conditions. While dietary interventions can play a role, they should be part of a broader therapeutic approach that considers all potential triggers and factors. Additionally, this highlights the significance of continuous research in the field to ascertain the nuanced impacts of diet on different health outcomes, thereby ensuring that dietary recommendations are both evidence-based and contextually relevant for specific conditions [45].

### 4.3. Anthropometric Outcomes and Body Mass Index

Concerning anthropometric characteristics, our umbrella review suggested limited evidence or an inconsistent association between adhering to the MD and anthropometric outcomes; in particular, we found an unclear consensus among the BMI changes. A recent systematic review published in 2023 aimed to clearly describe the effect of a randomized controlled trial lifestyle intervention based on MD promotion and BMI [19]. This recent systematic review stated a positive effect of adhering to the MD and a decrease in BMI and the percentage of obesity in Mediterranean countries. These results were not in line with previous systematic reviews [30,34] on this topic included in the present work. However, none of our included reviews provided a meta-analysis of RCTs, which could explain the discrepancy; moreover, most of the studies included in the review conducted by Iaccarino et al. [34] were cross-sectional and observational, in which it is not possible to infer cause–effect relationships. Jose Francisco Lopez-Gile al. [19], in their recent systematic review, proposed a possible explanation for how following the Mediterranean Diet (MD) contributes to sustaining a healthy body weight and averting obesity from an early age: primarily, the consumption of plant-based items, like fruits and vegetables, known for their large volume and low caloric density. Consequently, foods with a larger volume might take longer to consume compared to those with a smaller volume, potentially extending mealtimes, enhancing feelings of fullness, and decreasing overall calorie intake. Many fundamental components of the MD, like fruits, vegetables, whole grains, nuts, or seeds, which are fiber-rich, nutrient-dense, energy-poor foods, may promote consuming fewer calories. Moreover, specific compounds frequently used in the MD, like phenolic compounds in olive oil, omega-3 polyunsaturated fatty acids, vitamins, trace elements, and polyphenols, are recognized for their ability to regulate and preserve a healthy gut microbiome. They also enhance the integrity of the gut barrier, which is known to be compromised in cases of obesity and metabolic syndrome [46]. Finally, it is necessary to underline that the intrinsic variability in BMI measurements further increases the heterogeneity of the studies. Many studies included in the systematic review conducted by Iaccarino et al. [34] and Lassale et al. [27] use BMI as a self-reported measurement or verify BMI with weight and height, while other studies obtain BMI indirectly from skinfolds and circumferences.

### 4.4. Healthy Habits and Physical Fitness

With regards to health habits and PF outcomes, our analysis found suggestive evidence supporting the effectiveness of adherence to the Mediterranean Diet among PA levels, PF, and sedentary behavior. In particular, a direct association was found between the MD and PA levels; meanwhile, an inverse significant association was found for sedentary behaviors. The included studies confirmed that high levels of PA and fewer minutes spent engaged in sedentary behaviors are significantly correlated with greater adherence to MD.

Despite this, the measurement of PA levels and sedentary habits was very heterogeneous. Some studies used objective measures (i.e., accelerometers and pedometers), which are currently reliable tools for monitoring PA levels [47]. Others instead relied on self-reported questionnaires. Although the trend is positive and therefore suggests a clear association between PA and MD, future studies should take into consideration applying objective instruments and, when possible, including the methodology.

Regarding physical fitness, the three included systematic reviews and the meta-analysis confirmed a direct association with the MD and improved performance [27,31,33]. Very often, greater importance is given in the literature to PA levels; however, monitoring fitness status, in particular cardiorespiratory fitness, represents a fundamental proxy of health [48]. Recent studies suggested that children skipping breakfast have lower physical fitness [49]. Moreover, cardiorespiratory fitness assessed during a shuttle run test has been shown to be associated with healthy eating habits both in children [49] and adolescents [50], while healthy eating habits have been associated with children’s general fitness [51].

A limitation found in the included studies was that fitness status was sometimes monitored using the scientifically validated batteries (the Alfa battery Test and Eurofit) [28], while in other studies it was monitored by selecting only some motor tests, such as the 20 m shuttle run test [32,34].

### 4.5. Quality of Life and Pro-Inflammatory Biomarkers

Only one systematic review [24] confirmed the positive relationship between health-related quality of life and the MD using different questionnaires and scales to assess the QoL.

The Mediterranean Diet (MD) has long been recognized for its potential health benefits. A pivotal aspect of understanding these benefits lies in its relationship with clinical markers, particularly pro-inflammatory biomarkers, which play a significant role in various pathological conditions. The systematic review by Bujtor et al. [27] provides substantial evidence indicating an inverse association between adherence to the MD and pro-inflammatory biomarkers. Specifically, the biomarkers CRP, IL-6, and TNF were highlighted. Similar results are reported by Teixeira and colleagues [26]. These findings are essential for clinicians as they offer a potential dietary intervention to reduce inflammation, which is an underlying factor in many chronic diseases.

CRP, or C-reactive protein, is a well-documented marker for systemic inflammation. Elevated levels of CRP are often indicative of inflammation in the body, which can be a result of various factors, including certain diseases and conditions. An inverse association between MD adherence and CRP suggests that following the MD might contribute to lower systemic inflammation levels, potentially reducing the risk of diseases associated with chronic inflammation. Similarly, IL-6 and TNF are cytokines that play significant roles in the inflammatory response. The inverse relationship between MD adherence and these cytokines further strengthens the argument that the MD has anti-inflammatory properties. This is particularly crucial information for clinicians treating patients with inflammatory diseases, as dietary modifications could be one avenue for management or even prevention. However, it is important to approach these findings with a degree of caution. For instance, the results from Teixeira et al. [26] indicated a positive association between better adherence to the MD and lower blood CRP concentrations. Nevertheless, this finding was based on a singular cross-sectional study included in their review [52]. Hence, while the evidence is promising, there is a need for more longitudinal studies to substantiate these associations.

In conclusion, for clinicians, the potential anti-inflammatory properties of the MD, as suggested by its inverse association with key pro-inflammatory biomarkers, offer a compelling avenue for dietary recommendations. However, it is paramount that further research, particularly longitudinal studies, be conducted to validate and deepen our understanding of these associations. As always, dietary interventions should be considered in the broader context of individual patient needs and conditions [53,54].

### 4.6. Future Directions

Regarding cognitive functions in adulthood, there are meta-analyses providing convincing evidence for a protective effect of the MD in relation to neurodegenerative diseases, including, in particular, Alzheimer’s disease and dementia [3]. The relationship between the MD and cognitive functions was little investigated in children and adolescents and mainly based on cross-sectional studies. In particular, school age represents an important period for the development of executive functions, which are a set of important cognitive processes. Only a few studies (all cross-sectional), examined relationships between the MD and executive functions in childhood and adolescence. The HELENA (Healthy Lifestyle in Europe by Nutrition in Adolescence) study, which included 384 adolescents, investigated associations of three dietary patterns (the Diet Quality Index, the Ideal Diet Score, and the MD) with attention capacity. The results suggest that healthier dietary patterns, as indicated by a higher Diet Quality index and the Ideal Diet Score, were associated with higher attention capacity in adolescence; conversely, the MD was not associated [55]. More recent studies reported that MDs were positively associated with executive functions in school-age children. More specifically, the MD adherence score showed a positive association with selective attention and concentration of Chilean school children and with working memory of Italian primary school children [56,57]. In addition, positive associations between adherence to the MD and academic performance have been reported in Spanish and Greek students [58,59,60,61]. A systematic review [62] investigated the effects of dietary behaviors on the academic performance of school-aged children, including 41 studies, but only one concerned adherence to the MD. It was therefore not included in this umbrella review. In general, better adherence to healthier dietary patterns has been associated with better cognitive functions and academic achievement during school age. However, the effects of the MD on cognitive functions and academic achievement in young people need further investigation. In particular, long-term intervention studies to investigate the effects of improvements in diet quality on cognitive functioning are needed [63].

Moving to the concept of mental health, there is evidence to suggest that MD adherence may have a protective effect on mental health outcomes, but the vast majority of studies have been conducted in adults [64].

### 4.7. Limitations

In general, most of the included studies were observational, and in only two systematic reviews was a meta-analysis performed.

Unfortunately, limited longitudinal data are available regarding the effects of the MD on asthma or other health outcomes. Moreover, numerous studies in every review had already been included in other systematic reviews. In the included studies, it was found that adherence to the MD in children and adolescents was measured using different instruments, and some indices were adapted and modified from the original. Not all of these tools were developed specifically for use in children and adolescents. Furthermore, several tools have not been validated for the countries under study. Finally, information on food intake was obtained using different methods (food records, food frequency questionnaire, 24 h dietary recalls, etc.). However, MD adherence was mostly evaluated with the KIDMED index.

In recognizing the rich tapestry of cultural and dietary practices that define the broader Mediterranean region, we acknowledge the significance of embracing this diversity for a holistic understanding of the MD. Our umbrella review, while comprehensive in its scope, primarily focuses on the overarching principles and health outcomes associated with the Mediterranean Diet and does not delve into the distinctive dietary nuances inherent to each Mediterranean country, particularly those on the African and Middle Eastern shores. This limitation points to an important avenue for future research. We advocate for subsequent reviews and studies to undertake a more detailed exploration of the regional variations within the MDs. Moreover, while our review concentrates on the dietary components of the MD, we recognize the existence and importance of accompanying lifestyle practices. Future reviews might benefit from an integrated approach that encompasses both the dietary and lifestyle dimensions of the MD for a more holistic understanding of its health impacts.

## 5. Conclusions

The present umbrella review collected and evaluated all of the available studies on the impact of the MD on health outcomes in school-age children and adolescents from 6 to 19 years of age. Higher adherence to the MD in youth was found to be inversely associated with asthma, inflammation markers, and sedentary behavior and directly correlated with physical fitness, physical activity, and quality of life. There was limited evidence of the beneficial effect of the MD on maintaining a healthy body weight in childhood and no consensus on BMI. Finally, no systematic review was found for mental health, cardiovascular outcomes, cognitive functioning, or academic achievement. As the majority of the included studies were cross-sectional, future research with longitudinal and intervention designs in this target group is needed.

## Figures and Tables

**Table 1:** Inclusion/exclusion criteria based on PICO.

Parameter	Inclusion Criteria	Exclusion Criteria
Population	School-age children and adolescents of any gender and ethnicity from 6 to 19 years of age	We excluded children populations <6 years and >19 years
Intervention	Adherence to the Mediterranean Diet	Studies focused on different types of diet
Comparator	Any other type of diet	
Outcome	Physical and mental health outcomes, cognitive functioning, academic achievement, and quality of life	Other outcomes
Study design	Systematic review with or without meta-analysis written in English	Narrative reviews and scoping reviews were excluded

**Table 2:** Characteristics of the analyzed systematic reviews.

First Author, Year, Country	Type of Review; Number of Eligible Studies Included/Total Number of Studies Included in the Review Designs of Eligible Studies	Total sample Size of Eligible Studies Included/Total Sample Size of all Studies Included in the Review; Age Range and Countries of Participants of Included Studies	Adherence to the MD and Outcome Measures (Method of Assessment)	Assessment of Mediterranean Diet Adherence	Overall Results	Strengths of the Reviews	Limitations of the Reviews
Bujtor et al. [26] 2021 Australia, Spain, UK	Type of review: systematic review 10/53 included studies: 10 observational studies	Sample size = 5661/51,556 Age = 6.5–18 y Countries = Colombia, Portugal, Austria, Belgium, France, Germany, Greece, Hungary, Italy, Spain, Sweden, USA, Turkey	Inflammation markers: CRP, TNF-alpha, IL-1, IL-2, IL-6, TGF-Beta1, sVCAM1, IL-4, IL-17, IL-33	KIDMED; m-KIDMED; MDS; m-MDS; ad hoc score	MD and CRP Inverse significant association: 2 studies (only 1 in females) Direct significant association: 1 study (only in males) Non-significant association: 6 studies (1 in DMT1 patients and 3 in obese/overweight patients) MD and IL-1 Inverse significant association: 1 study Non-significant association: 1 study MD and IL-2 Inverse significant association: 1 study Non-significant association: 1 study MD and IL-6 Inverse significant association: 1 study Non-significant association: 3 studies (1 in DMT1 patients) MD and TNF-alpha Inverse significant association: 1 study Non-significant association: 3 studies MD and TGF-Beta1 Direct significant association: 1 study (only in males) MD and sVCAM1 Inverse significant association: 1 study MD and IL-4 Inverse significant association: 1 study Non-significant association: 2 studies MD and IL-17 Inverse significant association: 1 study MD and IL-33 Direct significant association: 1 study (only in asthmatics)	Adequate adherence to MD in healthy populations results in decreased levels of pro-inflammatory biomarkers	Heterogeneity of studies in terms of sample size, age of participants, biological markers considered, index for assessing MD adherence, health status of the populations Studies do not consider the biological effect of food (intake versus absorption) Currently no consensus regarding the inflammatory biomarkers best used to represent chronic low-grade inflammation in children and adolescents Biomarker measurement errors, such as sampling, storage, and laboratory errors, cannot be excluded
Eslami et al. [31] 2020 Iran	Type of study: systematic review 5/11 included studies: 5 cross-sectional	Sample size = 180,898/198,271 Age = 8–18 y Countries = Spain, Iceland, Chile, Greece	PF (20 m—SRT (stages); 20 m—SRT (VO2 max))	KIDMED	MD and PF Direct significant association: 5 studies	Eligible studies showed that MD adherence was directly associated with CRF improvement	Study designs (all cross-sectional) Did not control for major potential confounding factors (PA and total energy intake) Most studies were conducted with populations living in developed or high-income countries
García-Hermoso et al. [27] 2022 Spain, Chile	Type of review: systematic review with meta-analysis 39/39 included studies: 37 cross-sectional 2, longitudinal (only data from baseline)	Sample size = 565,421/565,421 Age = 6–19 y Countries = Chile, Colombia, Israel, Portugal, Spain, Greece, Iceland, Estonia, Italy, Lithuania, Croatia, Serbia	PA (instrument, i.e., actigraph, questionnaire) SB (screen media time or frequency; sitting time): PF: CRF, muscular fitness, speed—agility (Eurofit Battery, Alpha-Fitness Battery)	KIDMED	MD and PA (r = 0.14; 95% CI 0,11, 0,17; I2 88.6) MD and CRF (r = 0.22; 95% CI 0.13, 0.31; I2 95.7) MD and muscular fitness (r = 0.11; 95% CI 0.03, 0.18; I2 95.4) MD and speed—agility (r = –0.06; 95% CI –0.12, –0.01; I2 84.2) MD and SB (r = –0.15; 95% CI –0.20, –0.10; I2 97.3) MD and SB in children (r = –0.21; 95% CI –0.29, –0.12; I2 96.5) MD and CRF in adolescents (r = 0.30; 95% CI 0.12, 0.47; I2 96.5)	Weak-to-moderate direct relationships between MD adherence and PA, CRF, and muscular fitness Weak-to-moderate inverse relationship between MD adherence and SB and speed—agility Youths with higher adherence to the MD were more likely to be physically active and fit and have a less sedentary lifestyle	Heterogeneity between studies in the associations, exposure, outcomes assessment, and publication bias Study designs (cross-sectional) Most of the studies did not consider potential confounding factors, such as socio-economic status and/or parental education
Garcia-Marcos et al. 2013 [24] Spain, Chile, Germany, Greece	Type of study: systematic review with meta-analysis 7/8 included studies: 7 cross-sectional	Sample size = 38,047/39,804 Age = 6–18 y Countries = Spain, Mexico, Albania, China, Ecuador, Estonia, France, Georgia, Germany, Ghana, Greece, India, Italy, Latvia, The Netherlands, New Zealand, Norway, Sweden, Turkey, UK, West Bank	Asthma: current wheeze (CW), current severe wheeze (CSW), asthma ever (AE) (wheeze episodes survey)	KIDMED; MDS; m-MDS	MD and CW OR: 0.85; 95% CI 0.75–0.98; *p* = 0.02 Mediterranean centers: OR: 0.79; 95% CI 0.66–0.94; *p* = 0.009 Non-Mediterranean centers: OR: 0.91; 95% CI 0.78–1.05; *p* = NS MD vs. non-MD centers: Q = 1.38; *p* = NS MD and CSW OR: 0.82; 95% CI 0.55–1.22; *p* = NS Mediterranean centers: OR: 0.66; 95% CI 0.48–0.90; *p* = 0.008 Non-Mediterranean centers: OR: 0.99; 95% CI 0.79–1.25; *p* = NS MD vs. non-MD centers: Q: 4.33; *p* = 0.037 MD and AE OR: 0.86; 95% CI 0.78–0.95; *p* = 0.004 Non-Mediterranean centers: OR: 0.86; 95% CI 0.75–0.98; *p* = 0.027 Mediterranean centers: OR: 0.86; 95% CI 0.74–1.01; *p* = NS MD vs. non-MD regions: Q = 0.001; *p* = NS	Adherence to the MD is a protective factor for CW, SCW, and AE specifically for Mediterranean centers	Heterogeneity in adherence to the MD assessment Study designs (all studies were cross-sectional) The use of the highest vs. the lowest tertile instead of a different approach, such as using the median as a cut-off point, probably minimized the effect of the different scoring systems across studies
Iaccarino et al. [33] 2017 Italy	Type of study: systematic review 25/58 included studies: 23 cross-sectional, 2 longitudinal	Sample size = 51,781/137,846 Age = 6–19 y Countries = Spain, Italy, Greece, UK, Cyprus, Portugal, Ireland	Anthropometric variables/body composition: BMI, WC, BF PA (questionnaires, accelerometers) SB (media screen time) PF (20 m Shuttle Run test)	KIDMED; m-KIDMED; MDS; m-MDS	MD and BMI Inverse significant association: 8 studies Direct significant association: 1 study Non-significant association: 12 studies 2 prospective studies found no longitudinal relationship MD and WC Inverse significant association: 2 studies Direct significant association: 1 study Non-significant association: 4 studies MD and BF Non-significant association: 5 studies MD and PA Direct significant association: 14 studies Non-significant association: 3 studies MD and PF Direct significant association: 1 study MD and SB Inverse significant association: 9 studies Non-significant association: 1 study	Most of the eligible studies showed that MD adherence was directly associated with physical activity and inversely associated with sedentary behavior, while the results for weight status were not consistent	Study designs (most of the studies were cross-sectional) Methodological differences and limitations in the studies included Use of self-reported anthropometric data could have biased the association between MD adherence and weight status
Koumpagioti et al. [28] 2022 Greece	Type of study: systematic Review 7/12 included studies: 5 cross-sectional, 1 case-control, 1 cohort	Sample size = 33,340/34,972 Age = 6–19 y Countries = Turkey, Greece, Peru, Lebanon, France	Asthma (spirometry): physician-diagnosed asthma, ever asthma symptoms, current asthma, asthma control, FEV1, FVC, Fractional Exhaled Nitric Oxide (FeNO) Allergies: physician-diagnosed allergic rhinitis, lifetime rhinitis, current rhinoconjunctivitis, atopic status, current eczema	KIDMED; MDS; m-MDS	MD and asthma Inverse significant association: 4 studies Non-significant association: 1 study MD and allergies Non-significant association: 4 studies	Adherence to the MD seemed to have a protective role against childhood asthma, but no effect was found on allergic rhinitis, eczema, or atopy	Heterogeneity among the included studies in the designs, sample sizes, tools assessing MD adherence, participants’ ages, variable outcomes, and adjusted confounders Study designs (most of the studies were cross-sectional)
Lassale et al. [29] 2021 Spain	Type of study: systematic review 45/55 included studies: 6 RCTs, 36 cross-sectional, 3 longitudinal	Sample size = 234,236/601,740 Age: = 6–19 y Countries = Spain, Italy, Greece, Mexico, Israel, Chile, Colombia, Croatia, Lebanon, Estonia, Iceland, Iran, Lithuania, Serbia, USA, Finland, Turkey, UK	Anthropometric variables: BMI, WHtR, WC, general and abdominal obesity, BF	KIDMED; m-KIDMED; m-MDS; MediLIFE Index; Krece plus test	MD and obesity Intervention studies: Before/after comparisons: significant reduction: 4 studies With control group comparisons: Significant differences: 2 studies Non-significant differences: 2 studies Observational studies: General adiposity: Non-significant association: 25 studies Inverse association: 14 studies Abdominal adiposity: Non-significant association: 7 studies Inverse association: 2 studies	Most of the eligible studies showed limited evidence for the MD and obesity	Only one of these studies was of high quality and included paternal educational level as a potential confounder in the analysis Low quality of the included studies Heterogeneity in the adherence to the MD assessment Study design (most of the studies were cross-sectional)
Lv et al. [30] 2014 USA	Type of study: systematic review 10/31 included studies: 9 cross-sectional, 1 cohort	Sample size = 90,102/518,782 Age = 6–18 y Countries = Greece, Spain, Mexico, Ecuador, Estonia, France, Georgia, Germany, Ghana, Iceland, India, Italy, Latvia, New Zealand, Norway, Sweden, Turkey, UK	Asthma: asthma symptoms, current severe asthma, ever asthma, prevalence and severity of ever asthma, current occasional asthma, clinically significant asthma, ever wheeze, current wheeze, atopic wheeze, persistent wheeze, exercise wheeze, wheezing ever with atopy, lung function (FEV1, FVC), inflammatory response (IL-8)	KIDMED: m-KIDMED; MDS; m-MDS; ad hoc score	MD and asthma Inverse significant association: 7 studies Non-significant association: 3 studies	Higher adherence to the Mediterranean Diet may be associated with reduced asthma risk in children	Study designs (most of the studies were cross-sectional) Heterogeneity in MD adherence assessment and in asthma outcomes
Papamichael et al. [32] 2017 Australia	Type of study: systematic review 12/15 included studies: 9 cross-sectional, 1 case-control, 2 longitudinal (only baseline)	Sample size = 100,968/103,248 Age = 6–19 y Countries = Spain, Greece, Mexico, Turkey, Peru, Brazil, Albania, China, Ecuador, Estonia, France, Georgia, Germany, Ghana, India, Italy, Latvia, The Netherlands, New Zealand, Norway, Spain, Sweden, UK, West Bank	Asthma: (ISAAC respiratory questionnaire; spirometry): current asthma, ever asthma, overall lifetime prevalence of asthma, any asthma symptoms, current severe asthma (CSA), current occasional asthma (COA), doctor-diagnosed asthma, severe asthma, exercise-induced asthma, asthma control, night cough, BHR (hyper-responsiveness), FEV1, IL-8, FVC, exhaled nitric oxide level (eNO), exhaled breath condensate (EBC), ever wheeze, ever diagnosed wheeze, exercise wheeze, wheeze limiting speech, wheeze disturbing sleep, current wheeze, wheezing ever with atopy, wheeze in last 12 months, severe attacks of wheeze, persistent wheeze, atopic wheeze, atopy	KIDMED, MDS, m-MDS	MD and asthma Inverse significant association: 8 studies Inverse non-significant association: 2 studies Non-significant association: 1 study Direct significant association: 1 study	Adherence to the Mediterranean dietary pattern may reduce asthma symptoms in children (limited evidence)	Heterogeneity in study methodologies, age of participants, and sample size The majority of studies were cross-sectional
Romero Robles et al. [24] 2022 Peru	Type of study: systematic review 9/11 included studies: 9 cross-sectional	Sample size = 4654/6796 Age = 8–18 y Countries = Spain, Greece, Portugal, Lebanon, Italy	Health-related quality of life (KIDSCREEN-10, KIDSCREEN-27, KIDSCREEN-52, Peds-Ql)	KIDMED	MD and HRQoL general score Direct significant association: 5 studies Direct non-significant association: 2 studies Inverse non-significant association: 1 study MD and HRQoL subdomains All dimensions Direct significant association: 1 study Physical well-being and peers and school environment subdomain Direct significant association: 1 study	Positive correlation between adherence to the MD and HRQoL	Study designs (all studies were cross-sectional) Heterogeneity of the measurements used for HRQoL
Teixeira et al. [25] 2022 Portugal	Type of review: systematic review 23/128 included studies: 18 cross-sectional, 1 RCT, 1 case-control, 3 cohort longitudinal	Sample size = 34,266/329,898 Age = 6–17 y Countries = Italy, Spain, Portugal, Turkey, Chile, Greece, Colombia, Morocco, Germany, Austria, Belgium, France, Hungary, Sweden, UK, England	Anthropometric variables/body composition: BMI, WC, BF, skinfold thickness, neck circumference Clinical markers: albuminuria, blood pressure, C-RP, bone mineral density Asthma (symptoms) ADHD (diagnosis) Depression, night eating syndrome	KIDMED; m-KIDMED; MDS; m-MDS	MD and BMI Intervention studies: Inverse significant association: 1 study Observational studies: Inverse significant association: 6 studies Direct significant association: 1 study Non-significant association: 8 studies MD and WC Inverse significant association: 4 studies Direct significant association: 1 study Non-significant association: 1 study MD and BF Inverse significant association: 2 studies MD and subscapular skinfold thickness Inverse significant association: 1 study MD and neck circumference Inverse significant association: 1 study MD and albuminuria Inverse non-significant association: 1 study MD and blood Pressure: Inverse significant association: 1 study Direct significant association: 1 study Non-significant association: 1 study MD and C-RP Inverse significant association: 1 study MD and bone mineral density Direct significant association: 1 study MD and asthma Inverse significant association: 1 study MD and ADHD Inverse significant association: 1 study MD and depression Non-significant association: 1 study MD and night eating syndrome Non-significant association: 1 study	Inconsistent association between MD adherence and BMI Potential significant association between MD adherence and anthropometric variables that needs to be further investigated; the evidence is still scarce	Different characteristics of studies in terms of sample size, age of participants, outcomes assessment, method of food intake consumption Study designs (most of the studies were cross-sectional)

Abbreviations: MD = Mediterranean Diet; PA = physical activity; SB = sedentary behavior; CRF = cardiorespiratory fitness; PF = physical fitness; BMI= body mass index; WC = waist circumference; ST = skinfold thickness; BF= body fat; WHtR = waist to height ratio; CRP = C-reactive protein; FEV1 = Forced Expiratory Volume in 1 s; FVC = Forced Vital Capacity; KIDMED = Mediterranean Diet Quality Index for children and adolescents; m-KIDMED = KIDMED index modified; MDS = Mediterranean Diet Score; m-MDS = Mediterranean Diet Score modified; MediLIFE Index = Mediterranean lifestyle index.

**Table 3:** Quality assessment.

Authors	Study Design	Quality
Romero Robles et al. [24]	Systematic review	Low risk
Garcia Marcos et al. [25]	Systematic review with meta-analysis	Medium risk
Teixeira et al. [26]	Systematic review	Low risk
Bujtor et al. [27]	Systematic review	Medium risk
García-Hermoso et al. [28]	Systematic review with meta-analysis	Low risk
Koumpagioti et al. [29]	Systematic review	Medium risk
Lassale et al. [30]	Systematic review	Medium risk
Lv et al. [31]	Systematic review	Low risk
Eslami et al. [32]	Systematic review	Low risk
Papamichael et al. [33]	Systematic review	Low risk
Iaccarino et al. [34]	Systematic review	Low risk

## Data Availability

Data are contained within the article.

## Supplementary Materials

The following supporting information can be downloaded at: https://www.mdpi.com/article/10.3390/healthcare12040449/s1. Table S1: Risk of bias.

## References

[1] UNESCO—Mediterranean Diet n.d. https://ich.unesco.org/en/RL/mediterranean-diet-00884.

[2] Willett W.C., Sacks F., Trichopoulou A., Drescher G., Ferro-Luzzi A., Helsing E., Trichopoulos D. (1995). Mediterranean diet pyramid: A cultural model for healthy eating. Am. J. Clin. Nutr..

[3] Dinu M., Pagliai G., Casini A., Sofi F. (2018). Mediterranean diet and multiple health outcomes: An umbrella review of meta-analyses of observational studies and randomised trials. Eur. J. Clin. Nutr..

[4] Grosso G., Marventano S., Yang J., Micek A., Pajak A., Scalfi L., Galvano F., Kales S.N. (2017). A comprehensive meta-analysis on evidence of Mediterranean diet and cardiovascular disease: Are individual components equal?. Crit. Rev. Food. Sci. Nutr..

[5] Rees K., Takeda A., Martin N., Ellis L., Wijesekara D., Vepa A., Das A., Hartley L., Stranges S. (2019). Mediterranean-style diet for the primary and secondary prevention of cardiovascular disease. Cochrane Database Syst. Rev..

[6] Rosato V., Temple N.J., La Vecchia C., Castellan G., Tavani A., Guercio V. (2019). Mediterranean diet and cardiovascular disease: A systematic review and meta-analysis of observational studies. Eur. J. Nutr..

[7] Sofi F., Cesari F., Abbate R., Gensini G.F., Casini A. (2008). Adherence to Mediterranean diet and health status: Meta-analysis. BMJ.

[8] Sofi F., Macchi C., Abbate R., Gensini G.F., Casini A. (2014). Mediterranean diet and health status: An updated meta-analysis and a proposal for a literature-based adherence score. Public Health Nutr..

[9] Guasch-Ferré M., Willett W.C. (2021). The Mediterranean diet and health: A comprehensive overview. J. Intern. Med..

[10] Dernini S., Berry E.M., Serra-Majem L., La Vecchia C., Capone R., Medina F.X., Aranceta-Bartrina J., Belahsen R., Burlingame B., Calabrese G. (2017). Med Diet 4.0, the Mediterranean diet with four sustainable benefits. Public Health Nutr..

[11] Sostenibilità Fondazione Dieta Mediterr n.d. https://www.fondazionedietamediterranea.it/dieta/sostenibilita/.

[12] Russo G.L., Siani A., Fogliano V., Geleijnse J.M., Giacco R., Giampaoli S., Iacoviello L., Kromhout D., Lionetti L., Naska A. (2021). The Mediterranean diet from past to future: Key concepts from the second “Ancel Keys” International Seminar. Nutr. Metab. Cardiovasc. Dis..

[13] García-Fernández E., Rico-Cabanas L., Rosgaard N., Estruch R., Bach-Faig A. (2014). Mediterranean diet and cardiodiabesity: A review. Nutrients.

[14] Sawyer S.M., Afifi R.A., Bearinger L.H., Blakemore S.-J., Dick B., Ezeh A.C., Patton G.C. (2012). Adolescence: A foundation for future health. Lancet.

[15] Craigie A.M., Lake A.A., Kelly S.A., Adamson A.J., Mathers J.C. (2011). Tracking of obesity-related behaviours from childhood to adulthood: A systematic review. Maturitas.

[16] Vilarnau C., Stracker D.M., Funtikov A., da Silva R., Estruch R., Bach-Faig A. (2019). Worldwide adherence to Mediterranean Diet between 1960 and 2011. Eur. J. Clin. Nutr..

[17] García Cabrera S., Herrera Fernández N., Rodríguez Hernández C., Nissensohn M., Román-Viñas B., Serra-Majem L. (2015). KIDMED Test; Prevalence of Low Adherence to the Mediterranean Diet in Children and Young; A Systematic Review. Nutr. Hosp..

[18] Pulgaron E.R., Delamater A.M. (2014). Obesity and Type 2 Diabetes in Children: Epidemiology and Treatment. Curr. Diab. Rep..

[19] López-Gil J.F., García-Hermoso A., Sotos-Prieto M., Cavero-Redondo I., Martínez-Vizcaíno V., Kales S.N. (2023). Mediterranean Diet-Based Interventions to Improve Anthropometric and Obesity Indicators in Children and Adolescents: A Systematic Review with Meta-Analysis of Randomized Controlled Trials. Adv. Nutr..

[20] Farella I., Miselli F., Campanozzi A., Grosso F.M., Laforgia N., Baldassarre M.E. (2022). Mediterranean Diet in Developmental Age: A Narrative Review of Current Evidences and Research Gaps. Children.

[21] Aromataris E., Fernandez R., Godfrey C.M., Holly C., Khalil H., Tungpunkom P. (2015). Summarizing systematic reviews: Methodological development, conduct and reporting of an umbrella review approach. Int. J. Evid. Based Healthc..

[22] Aromataris E., Munn Z. (2020). JBI Manual for Evidence Synthesis.

[23] Hossain M.M., Nesa F., Das J., Aggad R., Tasnim S., Bairwa M., Ma P., Ramirez G. (2022). Global burden of mental health problems among children and adolescents during COVID-19 pandemic: An umbrella review. Psychiatry Res..

[24] Romero-Robles M.A., Ccami-Bernal F., Ortiz-Benique Z.N., Pinto-Ruiz D.F., Benites-Zapata V.A., Casas Patiño D. (2022). Adherence to Mediterranean diet associated with health-related quality of life in children and adolescents: A systematic review. BMC Nutr..

[25] Garcia-Marcos L., Castro-Rodriguez J.A., Weinmayr G., Panagiotakos D.B., Priftis K.N., Nagel G. (2013). Influence of Mediterranean diet on asthma in children: A systematic review and meta-analysis. Pediatr. Allergy Immunol. Off. Publ. Eur. Soc. Pediatr. Allergy Immunol..

[26] Teixeira B., Afonso C., Rodrigues S., Oliveira A. (2022). Healthy and Sustainable Dietary Patterns in Children and Adolescents: A Systematic Review. Adv. Nutr..

[27] Bujtor M., Turner A.I., Torres S.J., Esteban-Gonzalo L., Pariante C.M., Borsini A. (2021). Associations of Dietary Intake on Biological Markers of Inflammation in Children and Adolescents: A Systematic Review. Nutrients.

[28] García-Hermoso A., Ezzatvar Y., López-Gil J.F., Ramírez-Vélez R., Olloquequi J., Izquierdo M. (2022). Is adherence to the Mediterranean diet associated with healthy habits and physical fitness? A systematic review and meta-analysis including 565 421 youths. Br. J. Nutr..

[29] Koumpagioti D., Boutopoulou B., Moriki D., Priftis K.N., Douros K. (2022). Does Adherence to the Mediterranean Diet Have a Protective Effect against Asthma and Allergies in Children? A Systematic Review. Nutrients.

[30] Lassale C., Fitó M., Morales-Suárez-Varela M., Moya A., Gómez S.F., Schröder H. (2022). Mediterranean diet and adiposity in children and adolescents: A systematic review. Obes. Rev. Off. J. Int. Assoc. Study Obes..

[31] Lv N., Xiao L., Ma J. (2014). Dietary pattern and asthma: A systematic review and meta-analysis. J. Asthma Allergy.

[32] Eslami O., Zarei M., Shidfar F. (2020). The association of dietary patterns and cardiorespiratory fitness: A systematic review. Nutr. Metab. Cardiovasc. Dis..

[33] Papamichael M.M., Itsiopoulos C., Susanto N.H., Erbas B. (2017). Does adherence to the Mediterranean dietary pattern reduce asthma symptoms in children? A systematic review of observational studies. Public Health Nutr..

[34] Iaccarino Idelson P., Scalfi L., Valerio G. (2017). Adherence to the Mediterranean Diet in children and adolescents: A systematic review. Nutr. Metab. Cardiovasc. Dis..

[35] Serra-Majem L., Ribas L., Ngo J., Ortega R.M., García A., Pérez-Rodrigo C., Aranceta J. (2004). Food, youth and the Mediterranean diet in Spain. Development of KIDMED, Mediterranean Diet Quality Index in children and adolescents. Public Health Nutr..

[36] Trichopoulou A., Costacou T., Bamia C., Trichopoulos D. (2003). Adherence to a Mediterranean diet and survival in a Greek population. N. Engl. J. Med..

[37] Serra-Majem L., Aranceta J., Ribas-Barba L., Sangil-Monroy M., Perez-Rodrigo C. (2003). Crecimiento y desarrollo: Dimensión alimentaria y nutricional. El cribado del riesgo nutricional en pediatría. Validación del test rápido, Krece Plus y resultados en la población española. Crecim Desarro Estud Enkid Krece Plus.

[38] Katsagoni C.N., Psarra G., Georgoulis M., Tambalis K., Panagiotakos D.B., Sidossis L.S., EYZHN Study Group (2020). High and moderate adherence to Mediterranean lifestyle is inversely associated with overweight, general and abdominal obesity in children and adolescents: The MediLIFE-index. Nutr. Res..

[39] Zhang J., He M., Yu Q., Xiao F., Zhang Y., Liang C. (2023). The Effects of a Healthy Diet on Asthma and Wheezing in Children and Adolescents: A Systematic Review and Meta-Analysis. J. Asthma Allergy.

[40] Alwarith J., Kahleova H., Crosby L., Brooks A., Brandon L., Levin S.M., Barnard N.D. (2020). The role of nutrition in asthma prevention and treatment. Nutr. Rev..

[41] Papadopoulou A., Panagiotakos D.B., Hatziagorou E., Antonogeorgos G., Matziou V.N., Tsanakas J.N., Gratziou C., Tsabouri S., Priftis K. (2015). Antioxidant foods consumption and childhood asthma and other allergic diseases: The Greek cohorts of the ISAAC II survey. Allergol. Immunopathol..

[42] Calder P.C. (2006). n-3 polyunsaturated fatty acids, inflammation, and inflammatory diseases. Am. J. Clin. Nutr..

[43] Tosti V., Bertozzi B., Fontana L. (2018). Health Benefits of the Mediterranean Diet: Metabolic and Molecular Mechanisms. J. Gerontol. A Biol. Sci. Med. Sci..

[44] Zheng T., Yu J., Oh M.H., Zhu Z. (2011). The Atopic March: Progression from Atopic Dermatitis to Allergic Rhinitis and Asthma. Allergy Asthma Immunol. Res..

[45] Cámara M., Giner R.M., González-Fandos E., López-García E., Mañes J., Portillo M.P., Rafecas M., Domínguez L., Martínez J.A. (2021). Food-Based Dietary Guidelines around the World: A Comparative Analysis to Update AESAN Scientific Committee Dietary Recommendations. Nutrients.

[46] García-Montero C., Fraile-Martínez O., Gómez-Lahoz A.M., Pekarek L., Castellanos A.J., Noguerales-Fraguas F., Coca S., Guijarro L.G., García-Honduvilla N., Asúnsolo A. (2021). Nutritional Components in Western Diet Versus Mediterranean Diet at the Gut Microbiota-Immune System Interplay. Implications for Health and Disease. Nutrients.

[47] Zheng Y., Won W.-K., Guan X., Trost S. Physical Activity Recognition from Accelerometer Data Using a Multi-Scale Ensemble Method. Proceedings of the AAAI Conference on Artificial Intelligence.

[48] Ortega F.B., Ruiz J.R., Castillo M.J., Sjöström M. (2008). Physical fitness in childhood and adolescence: A powerful marker of health. Int. J. Obes..

[49] Sandercock G.R.H., Voss C., Dye L. (2010). Associations between habitual school-day breakfast consumption, body mass index, physical activity and cardiorespiratory fitness in English schoolchildren. Eur. J. Clin. Nutr..

[50] Kafatos A., Codrington C.A., Linardakis M. (2005). Obesity in Childhood: The Greek Experience. World Rev. Nutr. Diet.

[51] Edwards J.U., Mauch L., Winkelman M.R. (2011). Relationship of nutrition and physical activity behaviors and fitness measures to academic performance for sixth graders in a midwest city school district. J. Sch. Health.

[52] Arouca A.B., Meirhaeghe A., Dallongeville J., Moreno L.A., Lourenço G.J., Marcos A., Huybrechts I., Manios Y., Lambrinou C.-P., Gottrand F. (2020). Interplay between the Mediterranean diet and C-reactive protein genetic polymorphisms towards inflammation in adolescents. Clin. Nutr..

[53] Cena H., Calder P.C. (2020). Defining a Healthy Diet: Evidence for the Role of Contemporary Dietary Patterns in Health and Disease. Nutrients.

[54] Tapsell L.C., Neale E.P., Satija A., Hu F.B. (2016). Foods, Nutrients, and Dietary Patterns: Interconnections and Implications for Dietary Guidelines. Adv. Nutr..

[55] Henriksson P., Cuenca-García M., Labayen I., Esteban-Cornejo I., Henriksson H., Kersting M., Vanhelst J., Widhalm K., Gottrand F., Moreno L.A. (2017). Diet quality and attention capacity in European adolescents: The Healthy Lifestyle in Europe by Nutrition in Adolescence (HELENA) study. Br. J. Nutr..

[56] Caamaño-Navarrete F., Latorre-Román P.Á., Párraga-Montilla J., Jerez-Mayorga D., Delgado-Floody P. (2021). Selective Attention and Concentration Are Related to Lifestyle in Chilean Schoolchildren. Children.

[57] Masini A., Sanmarchi F., Kawalec A., Esposito F., Scrimaglia S., Tessari A., Scheier L.M., Sacchetti R., Dallolio L. (2023). Mediterranean diet, physical activity, and family characteristics associated with cognitive performance in Italian primary school children: Analysis of the I-MOVE project. Eur. J. Pediatr..

[58] Esteban-Cornejo I., Izquierdo-Gomez R., Gómez-Martínez S., Padilla-Moledo C., Castro-Piñero J., Marcos A., Veiga O.L. (2016). Adherence to the Mediterranean diet and academic performance in youth: The UP&DOWN study. Eur. J. Nutr..

[59] Chacón-Cuberos R., Zurita-Ortega F., Martínez-Martínez A., Olmedo-Moreno E.M., Castro-Sánchez M. (2018). Adherence to the Mediterranean Diet Is Related to Healthy Habits, Learning Processes, and Academic Achievement in Adolescents: A Cross-Sectional Study. Nutrients.

[60] Tapia-Serrano M.A., Esteban-Cornejo I., Rodriguez-Ayllon M., Vaquero-Solís M., Sánchez-Oliva D., Sánchez-Miguel P.A. (2021). Adherence to the Mediterranean diet and academic performance in adolescents: Does BMI status moderate this association?. Clin. Nutr..

[61] Vassiloudis I., Yiannakouris N., Panagiotakos D.B., Apostolopoulos K., Costarelli V. (2014). Academic performance in relation to adherence to the Mediterranean diet and energy balance behaviors in Greek primary schoolchildren. J. Nutr. Educ. Behav..

[62] Burrows T., Goldman S., Pursey K., Lim R. (2017). Is there an association between dietary intake and academic achievement: A systematic review. J. Hum. Nutr. Diet Off. J. Br. Diet Assoc..

[63] Barbosa A., Whiting S., Simmonds P., Scotini Moreno R., Mendes R., Breda J. (2020). Physical Activity and Academic Achievement: An Umbrella Review. Int. J. Environ. Res. Public Health.

[64] O’Neil A., Quirk S.E., Housden S., Brennan S.L., Williams L.J., Pasco J.A., Berk M., Jacka F.N. (2014). Relationship Between Diet and Mental Health in Children and Adolescents: A Systematic Review. Am. J. Public Health.

