# Supplementary material for: Adherence to the Mediterranean Diet in Children and Adolescents and Association with Multiple Outcomes: An Umbrella Review
Source: Healthcare (Basel). 2024 Feb 9;12(4):449. doi: 10.3390/healthcare12040449 (PMC10887852; doi:10.3390/healthcare12040449)
Supplement: Supplementary file 1 [file healthcare-12-00449-s001.zip › healthcare-2796269-supplementary.pdf]

Table S1 Risk of Bias Assessment

Authors	Item 1	Item 2	Item 3	Item 4	Item 5	Item 6	Item 7	Item 8	Item 9	Item 10	Item 11	Score	Overall
Romero Robles et al. [24]	1	1	1	1	1	1	1	1	NA	1	1	10	Low risk
Garcia Marcos et al. [25]	1	-	1	1	0	0	-	1	0	1	1	6	Medium risk
Teixeira et al. [26]	1	1	1	1	1	1	1	1	NA	1	1	10	Low risk
Bujtor et al. [27]	1	1	1	1	1	-	-	0	NA	1	1	7	Medium risk
García-Hermoso et al. [28]	1	1	1		1	-	1	1	1	1	1	10	Low risk
Koumpagioti et al. [29]	1	1	0	1	0	0	1	1	NA	0	1	6	Medium risk
Lassale et al. [30]	1	1	1	0	1	-	-	1	NA	-	1	6	Medium risk
LV et al. [31]	1	1	1	1	1	1	1	1	1	-	1	10	Low risk
Eslami et al. [32]	1	1	1	1	1	1	1	1	NA	1	1	10	Low risk
Papamichael et al. [33]	1	1	1	1	1	1	1	1	NA	1	1	10	Low risk
Iaccarino et al. [34]	1	-	1	1	1	1	1	1	NA	1	1	9	Low risk

NA: Not Applicable
